# The prognostic nutritional index represents a novel inflammation-nutrition-based prognostic factor for nasopharyngeal carcinoma

**DOI:** 10.3389/fnut.2023.1036572

**Published:** 2023-02-16

**Authors:** Yan-Ming Jiang, Shi-Ting Huang, Xin-Bin Pan, Jia-Lin Ma, Xiao-Dong Zhu

**Affiliations:** ^1^Department of Radiation Oncology, Guangxi Medical University Cancer Hospital, Nanning, China; ^2^Department of Oncology, Affiliated Wuming Hospital of Guangxi Medical University, Nanning, China

**Keywords:** nasopharyngeal carcinoma, PNI, prognosis, nomogram, LDH

## Abstract

**Purpose:**

This study explored the relationship between the prognostic nutritional index (PNI) and overall survival rate (OS) in patients with nasopharyngeal carcinoma (NPC), and established and validated an effective nomogram to predict clinical outcomes.

**Methods:**

This study included 618 patients newly diagnosed with locoregionally advanced NPC. They were divided into training and validation cohorts at a ratio of 2:1 based on random numbers. The primary endpoint of this study was OS, progression-free survival (PFS) was the second endpoint. A nomogram was drawn from the results of multivariate analyses. Harrell’s concordance index (C-index), area under the receiver operator characteristic curve (AUC), and decision curve analysis (DCA) were used to evaluate the clinical usefulness and predictive ability of the nomogram and were compared to the current 8th edition of the International Union Against Cancer/American Joint Committee (UICC/AJCC) staging system.

**Results:**

The PNI cutoff value was 48.1. Univariate analysis revealed that age (*p* < 0.001), T stage (*p* < 0.001), N stage (*p* = 0.036), tumor stage (*p* < 0.001), PNI (*p* = 0.001), lymphocyte-neutrophil ratio (NLR, *p* = 0.002), and lactate dehydrogenase (LDH, *p* = 0.009) were significantly associated with OS, age (*p* = 0.001), T-stage (*p* < 0.001), tumor stage (*p* < 0.001), N-stage (*p* = 0.011), PNI (*p* = 0.003), NLR (*p* = 0.051), and LDH (*p* = 0.03) were significantly associated with PFS. Multivariate analysis showed that age (*p* < 0.001), T-stage (*p* < 0.001), N-stage(*p* = 0.02), LDH (*p* = 0.032), and PNI (*p* = 0.006) were significantly associated with OS, age (*p* = 0.004), T-stage (<0.001), N-stage (<0.001), PNI (*p* = 0.022) were significantly associated with PFS. The C-index of the nomogram was 0.702 (95% confidence interval [CI]: 0.653–0.751). The Akaike information criterion (AIC) value of the nomogram for OS was 1142.538. The C-index of the TNM staging system was 0.647 (95% CI, 0.594–0.70) and the AIC was 1163.698. The C-index, DCA, and AUC of the nomogram demonstrated its clinical value and higher overall net benefit compared to the 8th edition of the TNM staging system.

**Conclusion:**

The PNI represents a new inflammation-nutrition-based prognostic factor for patients with NPC. In the proposed nomogram, PNI and LDH were present, which led to a more accurate prognostic prediction than the current staging system for patients with NPC.

## 1. Introduction

Nasopharyngeal carcinoma (NPC) is highly prevalent in southern China and Southeast Asia, with an incidence rate of 20-30/100,000/year in some areas (1). Radiotherapy (RT) is the most important treatment for RT. Owing to the use of intensity-modulated radiotherapy (IMRT), the cure rate has significantly improved, particularly in terms of local recurrence-free and overall survival (OS). However, the incidence of distant metastasis remains high and is the main failure mode (2). Although the tumor lymph node metastasis (TNM) staging system is often considered the most valuable prognostic factor for NPC in clinical practice, the heterogeneity of patients at the same stage, who often have different risk factors, limits the ability of the system to differentiate between patients with different prognoses and make accurate treatment choices. Personalized and accurate predictions are challenging. Studies have shown a significant link between inflammatory markers and poor prognosis in patients with various types of tumors (3, 4). Thus, indicators of systemic inflammation, such as the neutrophil-to-lymphocyte ratio (NLR), platelet-to-lymphocyte ratio (PLR), and lymphocyte-to-monocyte ratio (LMR), have received increased attention (5–7). Recent studies have demonstrated a close relationship between immunonutritional status and tumor prognosis (8). The choice of treatment and quality of life are affected by patient nutritional status and immune function. Therefore, monitoring the nutritional and immune status of the body plays an important role in determining curative effects and prognosis (9). Hence, this study aimed to determine the value of inflammatory immune and nutritional indicators in the prognosis of patients with NPC.

Buzey first proposed the prognostic nutritional index (PNI) which is calculated from the serum albumin concentration and peripheral blood lymphocyte count (10). The PNI has been shown to be a valid indicator of a patient’s immune and nutritional status (11). The PNI was designed to assess the periodic operational immunity status and surgical risk in patients undergoing gastrointestinal surgery (12). Although investigators have demonstrated the significant value of PNI in the survival prognosis of many types of malignancy (13–15), few studies have reported the PNI prognosis of NPCs. While several studies have investigated the PNI in metastatic NPC (16, 17), the patients were treated using various regimens. Thus, the association of the PNI with the prognosis of survival, especially in patients with locally advanced NPC undergoing concurrent chemoradiotherapy (CCRT) with or without adjuvant chemotherapy (AC) remains unknown. Therefore, to eliminate therapeutic heterogeneity, the present study investigated the prognostic value of PNI in patients with locally advanced NPC.

## 2. Materials and methods

### 2.1. Patients

**Figure 1:**
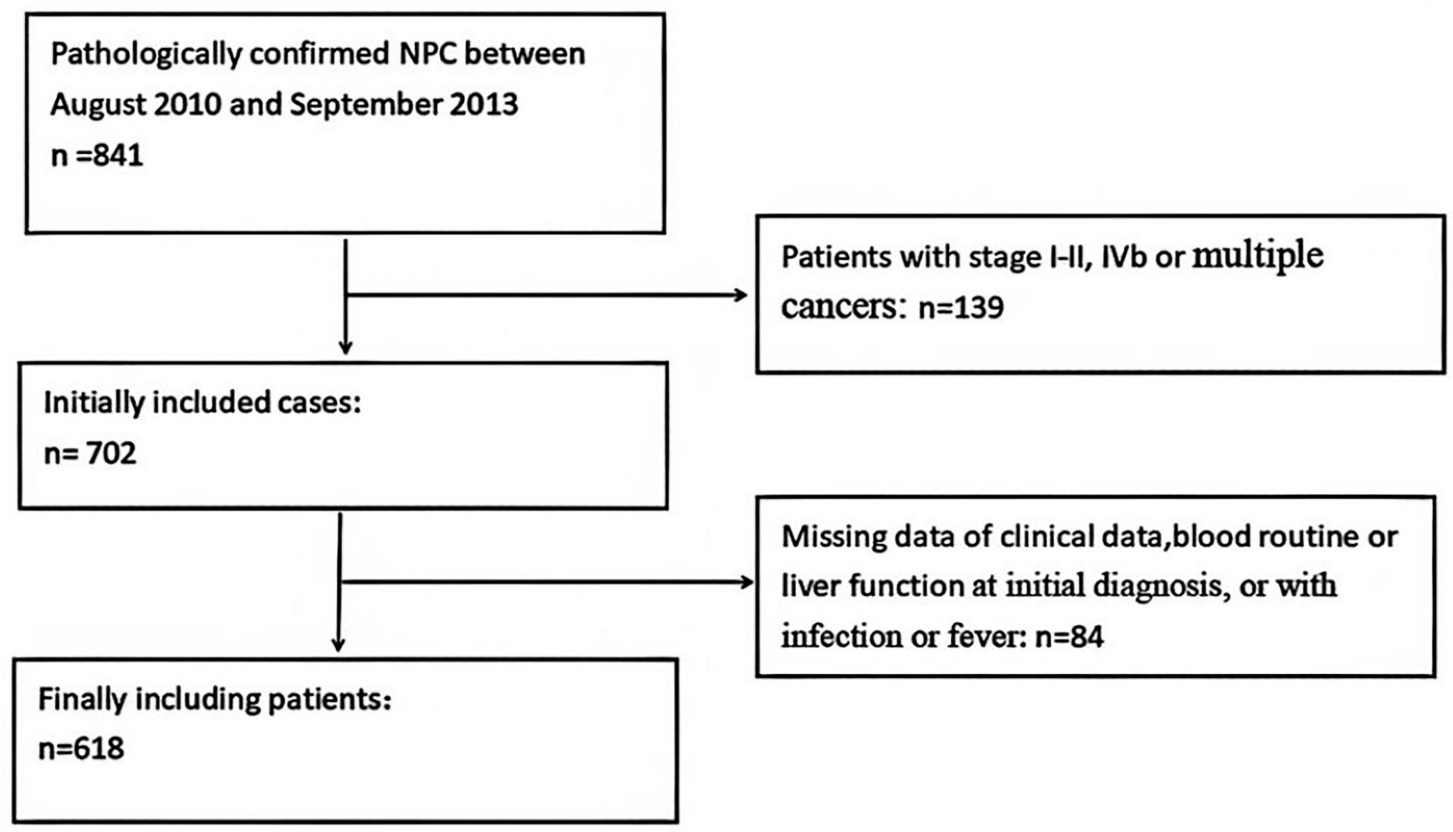
Flow chart of patient inclusion.

Data on patients with NPC who received chemoradiotherapy at Guangxi Medical University Cancer Hospital from 2010 to 2013 were collected and analyzed. NPC was diagnosed based on histological evidence. The inclusion criteria were: (1) patients with NPC who underwent IMRT, (2) received concurrent chemoradiation combined with or without adjuvant chemotherapy, and (3) had no hematology disease, infection, or hyperpyrexia. The exclusion criteria were: (1) multiple cancers at diagnosis, (2) TNM stages I–II and IVb, and (3) missing values in the relevant predictors or follow-up data (Figure 1).

The collected clinical data included age, sex, chemotherapy, clinical stage, radiotherapy dose, pretreatment lymphocyte count, pretreatment neutrophil count, pretreatment lactate dehydrogenase (LDH) level, pretreatment platelet count, serum albumin count (ALB), and pretreatment body mass index (BMI), which were calculated for each patient. The neutrophil-to-lymphocyte ratio (NLR) was calculated. The PNI was determined using the formula PNI = ALB+5 × lymphocyte count (g/L). This study was approved by the Ethics Committee of Guangxi Medical University Cancer Hospital (LW2022029). All data were anonymized; therefore, the requirement to obtain informed consent was waived.

### 2.2. Radiotherapy

Radical IMRT was performed in all patients. Primary nasopharyngeal gross tumor volume (GTVnx) and cervical lymph node tumor volume (GTVnd) were defined as tumors visible on computed tomography (CT), magnetic resonance imaging (MRI), 18F-fluorodeoxyglucose positron emission tomography with computed tomography (PET-CT), and physical examinations. Clinical target volume (CTV1) was determined using the formula CTV1 = GTVnx + (5–10 mm) margins. The CTV2 = CTV1 + (5–10 mm margins) included the GTVnd lymphatic region. The planning target volume (PTV) was calculated as PTV = CTV + 3 mm margins. The prescription dosage was: PGTVnx 70–75.9 Gy/31–32f, PGTVnd 60–73.6 Gy/30–32f, PCTV1 60–68 Gy/30 ~ 31f, and PCTV2 54–57.6 Gy/30–31 f.

### 2.3. Chemotherapy

All patients were administered concurrent chemotherapy. The chemotherapy regimen comprised cisplatin (2–3 cycles of 80–100 mg/m^2^ every 21 days). The adjuvant chemotherapy (AC) regimens included PF (cisplatin,80 mg/m^2^+ 5-fluorouracil 3,000 mg/m^2^), TPF (docetaxel 60 mg/m^2^ + cisplatin 60 mg/m^2^ + 5-fluorouracil 3,000 mg/m^2^), and TP (docetaxel 75 mg/m^2^ + cisplatin 75 mg/m^2^, every 21 days) after CCRT, for at least one cycle.

### 2.4. Patient follow-up

Patients were reviewed every 3 months for 2 years after the end of treatment. Within 3–5 years of the end of treatment, the reviews were performed every 6 months. Five years after the end of treatment, an annual review was conducted. OS was defined as the primary endpoint and was measured from the date of diagnosis to the date of death or the last follow-up, whichever occurred first.

### 2.5. Statistical analysis

To assess the sensitivity and specificity of OS, we used X-tile software (version 3.6.1, Yale University 2003-05) to determine the optimal cutoff values for the PNI and NLR (18). We evaluated other clinicopathological variables associated with the risk of progression, including BMI (19), age, and LDH (20), based on clinical significance, basic theoretical knowledge, and predictors identified in previous studies.

For continuous variables, means ± SD or medians are reported. Mann–Whitney U or Kruskal–Wallis tests were used for analysis, as appropriate. Numbers and proportions were reported for categorical variables. *χ*^2^ or Fisher’s exact tests were used for the analysis. Cox proportional hazards models were used for univariate and multivariate analyses. All *p*-values were two-tailed, with *p* < 0.05 considered statistically significant. IBM SPSS Statistics, version 23.0 (IBM Corp., Armonk, NY, United States) was used for statistical analysis.

Based on the results of the multivariate analysis, we established a nomogram. The predictive accuracy and discriminative ability were used to evaluate the predicted nomogram values. The C-index and calibration plot of the nomogram for 5-year OS were used to evaluate its performance. Harrell’s C-index and calibration plots were used for the external validation of the nomogram, which were compared to the current 8th edition of the International Union Against Cancer/American Joint Committee (UICC/AJCC) staging system based on C-index, receiver operating characteristic (ROC), and decision curve analysis (DCA). All steps were implemented using R software version 4.1.2 (R Foundation for Statistical Computing, Vienna, Austria).

## 3. Results

### 3.1. Patient characteristics

A total of 618 patients were enrolled and randomly assigned to the training and validation cohorts in a 2:1 ratio based on random numbers. The patient characteristics are shown in Table 1. No significant differences were observed between the training and validation cohorts (Table 1).

**Table 1:** Baseline patient characteristics.

Characteristic	Primary cohort (no. of patients)	Validation cohort (no. of patients)	*p* value
**No.**	412	206	
**Age**			0.676
<50 years	271	132	
≥50 years	141	74	
**Sex**			0.111
Male	320	148	
Female	92	58	
**Stage**			0.522
III	245	128	
IV	167	78	
**T-stage**			0.363
T1	7	7	
T2	89	51	
T3	193	95	
T4	123	53	
**N-stage**			0.464
N0	10	2	
N1	114	50	
N2	224	119	
N3	64	35	
**Adjuvant chemotherapy**			0.332
Yes	215	116	
No	197	90	
**WBC**			
Mean + SD	7.02 + 1.88	6.99 + 1.83	0.838
**N**			
Mean + SD	4.45 + 1.63	4.32 + 1.57	0.358
**L**			
Mean + SD	1.92 + 0.61	1.98 + 0.61	0.196
**PLT**			
Mean + SD	253.02 + 68.91	263.54 + 70.64	0.076
**NLR**			
Mean + SD	2.54 + 1.30	2.40 + 1.30	0.198
**LDH**			
Mean + SD	186.04 + 55.24	184.97 + 45.64	0.811
**ALB**			
Mean + SD	43.75 + 4.29	43.70 + 4.07	0.906
**PNI**			
Mean + SD	53.32 + 5.46	53.62 + 5.24	0.524

### 3.2. Survival outcomes

The median follow-up duration was 84 (range, 2–125) months. Of the patients, 148 died and 179 had advanced disease at the last follow-up. The 1-, 3-, and 5-year estimated OS rates in all cohorts were 98.5, 89.5, and 82.6%, respectively. The 1-, 3-, and 5-year estimated OS rates in the training cohort were 98.5, 88.3, and 80.9%, respectively, whereas those in the validation cohort were 98.5, 89.4, and 82.9%, respectively.

### 3.3. Prognostic factors

**Figure 2:**
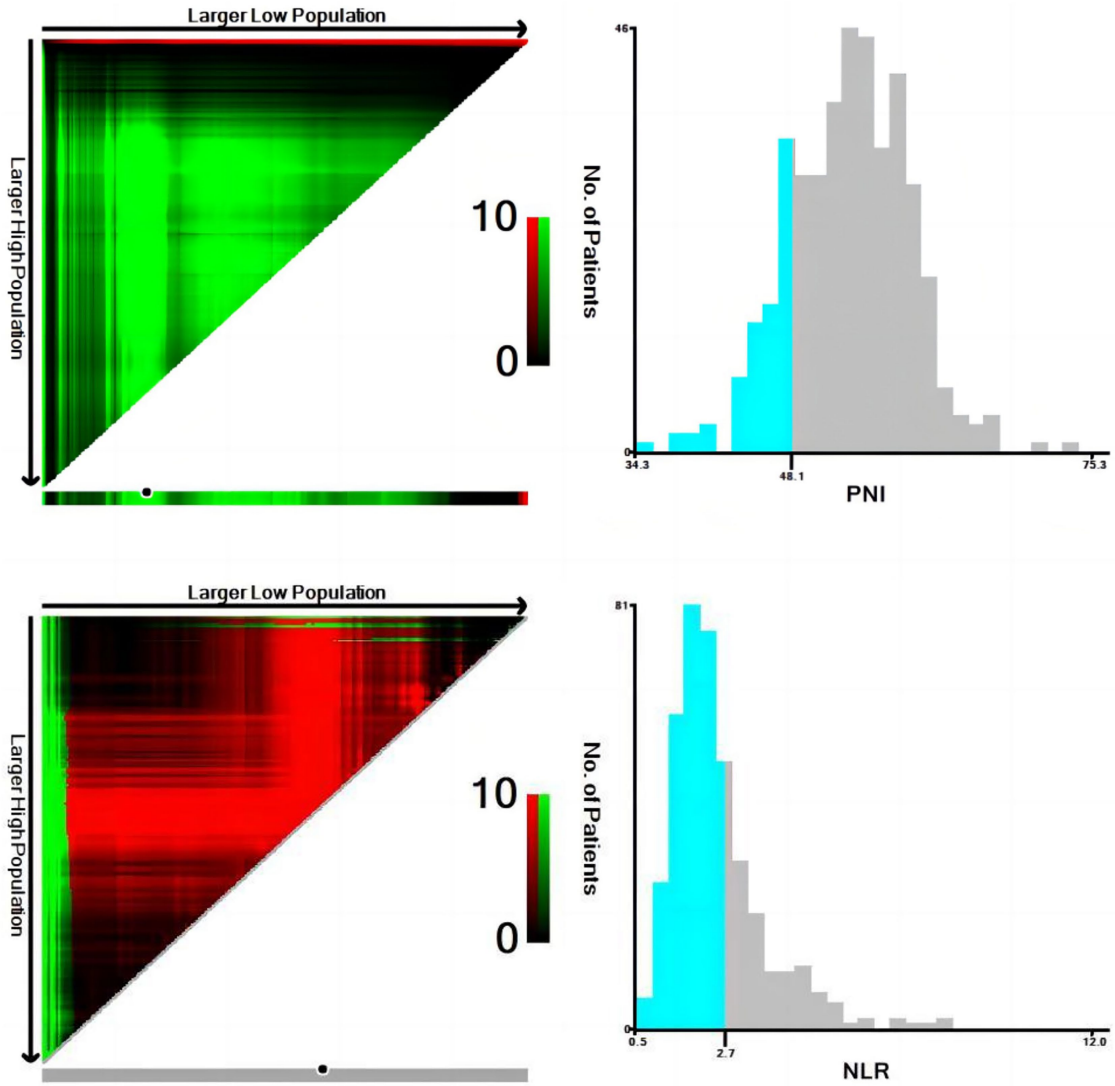
Calculation of optimal cut-off values of neutrophil-to-lymphocyte ratio (NLR) and prognostic nutritional index (PNI) by X-tile software.

NLR and PNI were used as test variables and OS as state variables. The X-tile program was used to determine the optimal cutoff values of NLR and PNI (2.7 and 48.1, respectively) (Figure 2). The PNI was significantly associated with age and T stage (Table 2). These findings are similar to those of related studies (21, 22).

**Table 2:** Baseline characteristics of patients grouped by PNI.

Characteristic	PNI < 48.1 (no. of patients)	PNI ≥ 48.1 (no. of patients)	*p* value
**Age**			0.012
<50 years	39	233	
≥50 years	34	106	
**Sex**			0.404
Male	54	266	
Female	19	73	
**Stage**			0.370
III	40	205	
IV	33	134	
**T-stage**			0.025
T1/2	9	81	
T3	30	149	
T4	34	109	
**N-stage**			0.574
N0/1	25	99	
N2	39	185	
N3	9	55	

**Figure 3:**
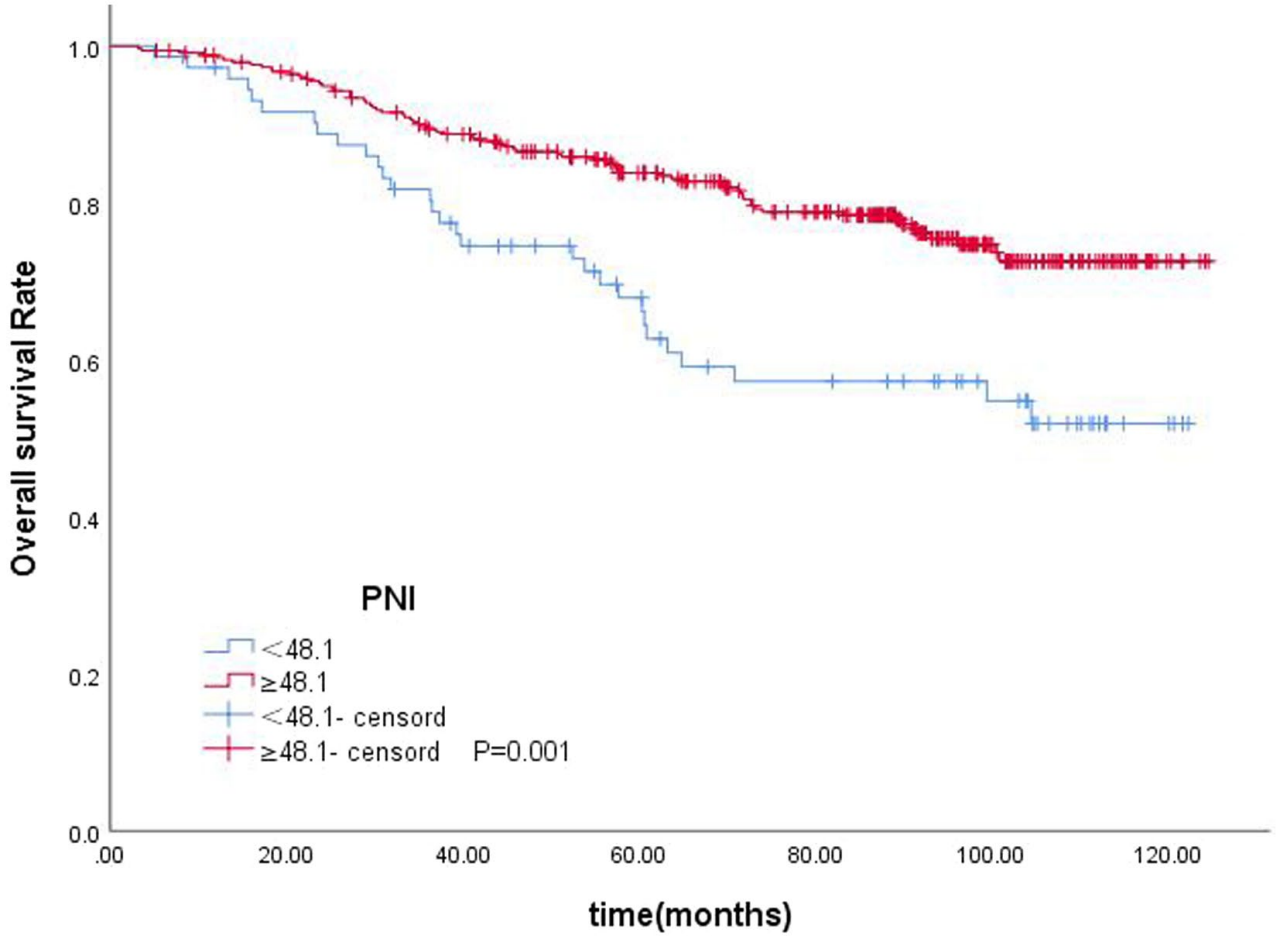
Kaplan–Meier curves for OS according to PNI.

**Figure 4:**
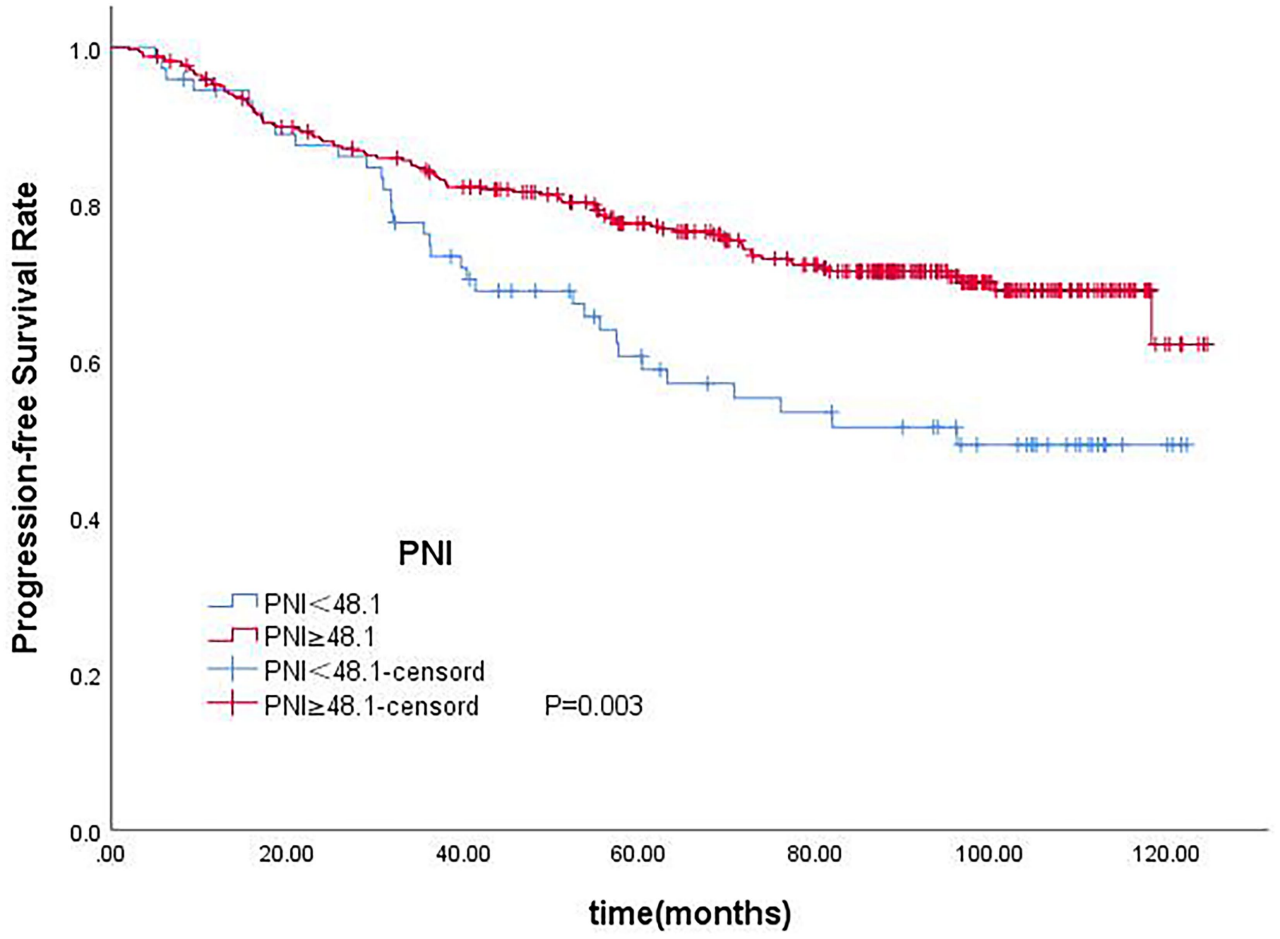
Kaplan–Meier curves for PFS according to PNI.

In the univariate analysis, age (*p* < 0.001), T-stage (*p* < 0.001), tumor stage (*p* < 0.001), N stage (*p* = 0.011), PNI (*p* = 0.001), NLR (*p* = 0.002), and LDH (*p* = 0.009) were significantly associated with OS. Age (*p* = 0.001), T-stage (*p* < 0.001), tumor stage (*p* < 0.001), N-stage (*p* = 0.011), PNI (*p* = 0.003), NLR (*p* = 0.051), and LDH (*p* = 0.03) were significantly associated with PFS. In the multivariate analysis, age (*p* < 0.001), T-stage (*p* < 0.001), N-stage (*p* = 0.020), PNI (*p* = 0.006), and LDH (*p* = 0.032) were significantly associated with OS. Age (*p* = 0.004), T-stage (<0.001), N-stage (<0.001), PNI (*p* = 0.022) were significantly associated with PFS (Tables 3, 4). PNI was an independent prognostic factor for both OS and PFS in nasopharyngeal carcinoma patients (Figures 3, 4).

**Table 3:** Identification of risk factors of overall survival (OS) by univariate and multivariate Cox models.

Variable	Number (%)	Univariate		Multivariate	
		*p* value	HR (95%CI)	*p* value	HR (95%CI)
**Age**					
<50 year	271 (65.8%)		Ref		Ref
≥50 year	141 (34.2%)	<0.001	2.31 (1.57–3.39)	<0.001	2.14 (1.44–3.18)
**Gender**					
Female	92 (22.3%)		Ref		
Male	320 (77.7%)	0.167	1.42 (0.86–2.34)		
**T-stage**					
T1/2	96 (23.3%)	<0.001	Ref	<0.001	Ref
T3	193 (46.8%)	0.109	1.62 (0.89–2.91)	0.077	1.74 (0.94–3.19)
T4	123 (29.9%)	<0.001	3.06 (1.71–5.49)	<0.001	3.14 (1.71–5.77)
**N-stage**					
N0/1	124 (30.1%)	0.036	Ref	0.030	Ref
N2	224 (54.4%)	0.072	1.55 (0.96–2.51)	0.040	2.10 (1.27–3.45)
N3	64 (15.5%)	0.011	2.19 (1.19–3.99)	0.020	2.69 (1.46–4.98)
**Stage**					
III	245 (59.5%)		Ref		Ref
IV	167 (40.5%)	<0.001	2.42 (1.64–3.57)	0.288	1.76 (0.62–4.98)
**NLR**					
<2.7	277 (67.2%)		Ref		Ref
≥2.7	135 (32.8%)	0.002	1.85 (1.25–2.72)	0.350	1.23 (0.79–1.89)
**LDH**					
<240	369 (89.6%)		Ref		Ref
≥240	43 (10.4%)	0.009	1.97 (1.19–3.28)	0.032	1.76 (1.05–2.95)
**PNI**					
<48.1	73 (17.7%)		Ref		Ref
≥48.1	339 (82.3%)	0.001	0.47 (0.31–0.72)	0.006	0.54 (0.35–0.84)
**BMI**					
<18	23 (5.6%)	0.520	Ref		
18–24	254 (61.9%)	0.293	0.67 (0.32–1.41)		
>24	134 (32.5%)	0.259	0.64 (0.29–1.39)		
AC					
Yes	197 (47.8%)		Ref		
No	215 (52.2%)	0.330	0.824 (0.59–1.22)		

**Table 4:** Identification of risk factors of PFS by univariate Cox models and multivariate Cox models.

Variable	Number(%)	Univariate		Multivariate	
		*p* value	HR (95%CI)	*p* value	HR (95%CI)
**Age**					
<50 year	271 (65.8%)		Ref		Ref
≥50 year	141 (34.2%)	<0.001	1.83 (1.29–2.60)	0.004	0.77 (0.64–0.92)
**Gender**					
Female	92 (22.3%)		Ref		
Male	320 (77.7%)	0.118	1.43 (0.91–2.25)		
**T-stage**					
T1/2	96 (23.3%)	<0.001	Ref	<0.001	Ref
T3	193 (46.8%)	<0.001	0.35 (0.21–0.59)	0.001	0.58 (0.41–0.81)
T4	123 (29.9%)	<0.001	0.49 (0.33–0.72)	0.66	0.94 (0.73–1.22)
**N-stage**					
N0/1	124 (30.1%)	0.003	Ref	<0.001	Ref
N2	224 (54.4%)	0.001	0.38 (0.22–0.66)	<0.001	0.51 (0.37–0.69)
N3	64 (15.5%)	0.079	0.67 (0.43–1.05)	0.192	1.18 (0.92–1.50)
**Stage**					
III	245 (59.5%)		Ref		Ref
IV	167 (40.5%)	<0.001	2.33 (1.64–3.32)	0.847	1.09 (0.45–2.67)
**NLR**					
<2.7	277 (67.2%)		Ref		Ref
≥2.7	135 (32.8%)	0.049	1.43 (0.99–2.04)	0.881	1.03 (0.69–1.53)
**LDH**					
<240	369 (89.6%)		Ref		Ref
≥240	43 (10.4%)	0.030	1.70 (1.05–2.75)	0.168	1.41 (0.86–2.31)
**PNI**					
<48.1	73 (17.7%)		Ref		Ref
≥48.1	339 (82.3%)	0.003	0.55 (0.37–0.82)	0.022	0.62 (0.41–0.93)
**BMI**					
<18	23 (5.6%)	0.755	Ref		
18–24	254 (61.9%)	0.457	1.32 (0.64–2.72)		
>24	134 (32.5%)	0.862	1.04 (0.71–1.52)		
AC					
Yes	197 (47.8%)		Ref		
No	215 (52.2%)	0.531	0.89 (0.63–1.27)		

### 3.4. Nomogram development and validation

**Figure 5:**
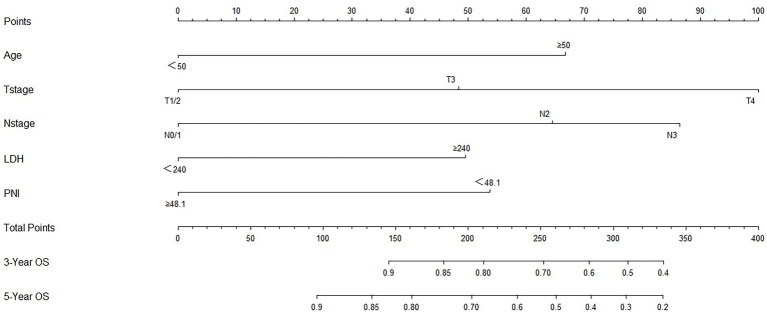
Prognostic nomogram of survival probabilities at 3- and 5-year in patients with nasopharyngeal carcinoma (NPC).

**Figure 6:**
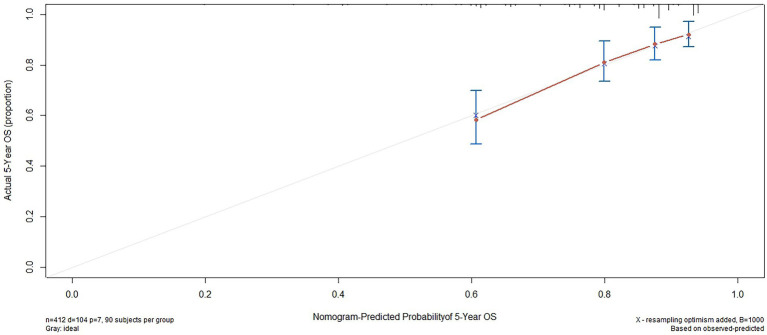
Calibration plots of survival probabilities at 5-year in patients with NPC.

**Figure 7:**
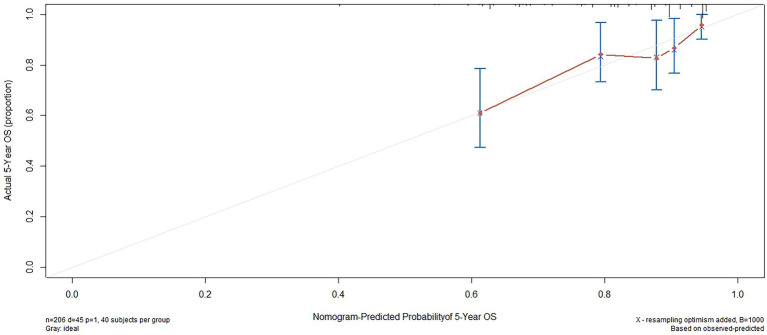
External validation of the nomogram to predict 5-year OS likelihoods in patients with NPC in the validation cohort.

Based on the results of the multivariate analysis, a nomogram was constructed for predicting OS by the ‘survival’ and ‘rms’ packages in R 4.1.2 (Figure 5). Each variable subtype was assigned a score based on a score table. To predict the 3-and 5-year OS, we summed the scores for each variable, obtained an overall score, and calculated the overall survival. The C-index of the nomogram was 0.702 (95% confidence interval [CI], 0.653–0.751). The Akaike information criterion (AIC) value of the nomogram for OS was 1142.538. Excellent agreement was observed in the calibration graphs between the nomogram-predicted probabilities and actual observations of the 5-year OS (Figure 6). To confirm the accuracy of the nomogram in the validation cohort, the C-index and calibration slope were applied. The C-index was 0.719 (95% CI, 0.580–0.858). The calibration curves for the validation cohort showed optimal agreement between the actual observations and the nomogram prediction of 5-year OS (Figure 7).

### 3.5. Predictive accuracy for OS compared between nomogram and TNM staging system

**Figure 8:**
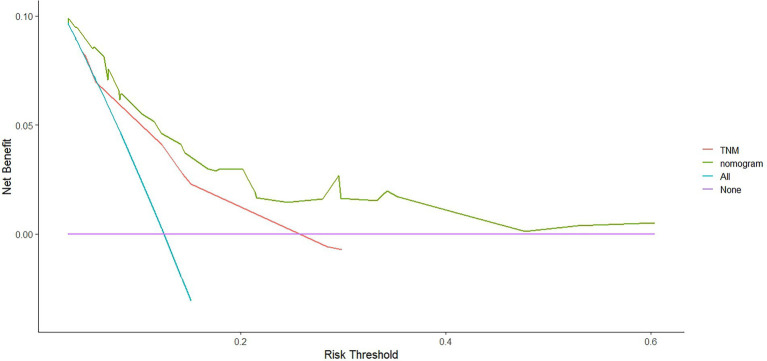
The decision curves (DCA) of OS at 3-year by the nomogram and the TNM staging system.

**Figure 9:**
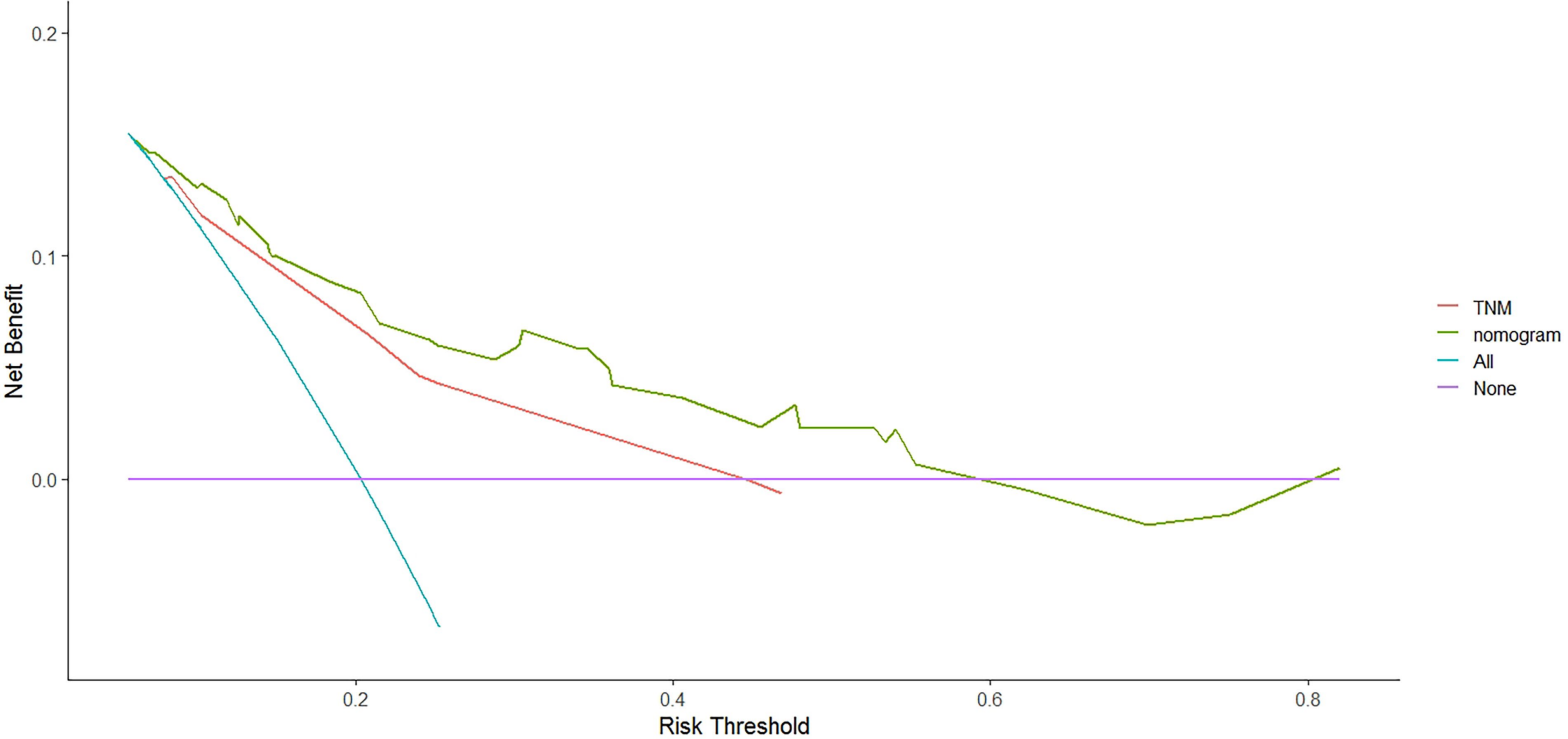
The decision curves (DCA) of OS at 5-year by the nomogram and the TNM staging system.

**Figure 10:**
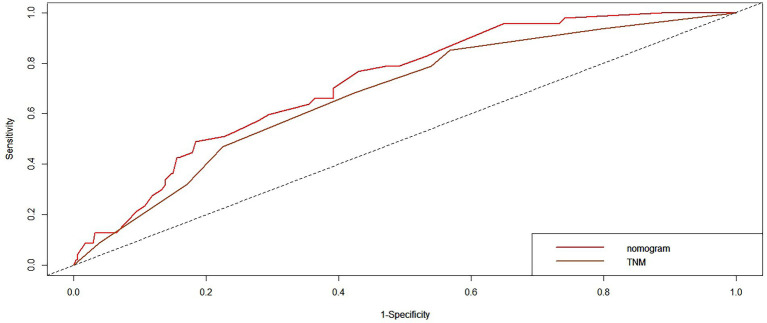
The time-dependent receiver operating characteristic (ROC) curves of OS at 3-year by the nomogram and the TNM staging system.

**Figure 11:**
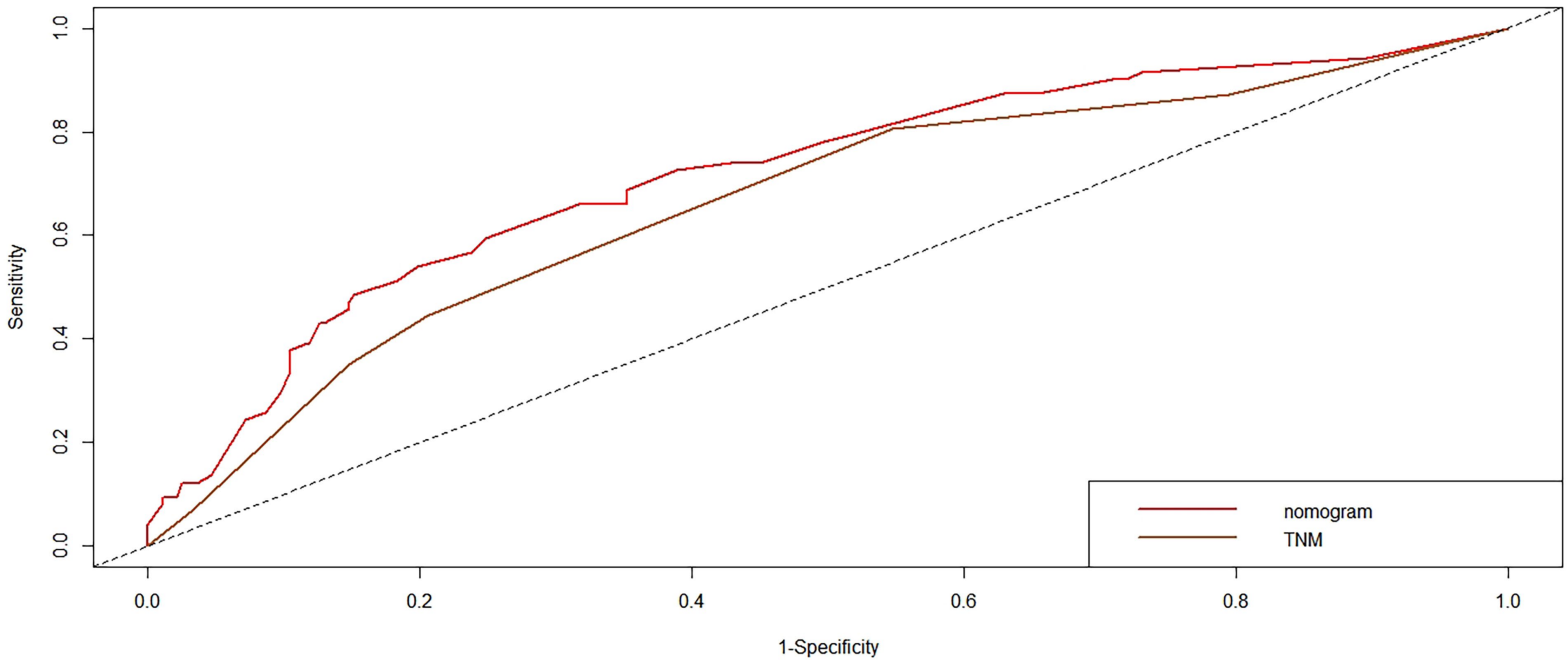
The time-dependent ROC curves of OS at 5-year by the nomogram and the TNM staging system.

We compared the accuracy of the prediction of 5-year OS between the nomogram and the TNM staging system. The C-index and AIC of the TNM staging system were 0.647 (95% CI, 0.594–0.70) and 1163.698, respectively, while those for the nomogram were 0.702 and 1142.538, respectively. Furthermore, the results of the DCA and time-dependent ROC analysis also indicated a higher net benefit of the clinical application of the nomogram in predicting OS compared to the 8th edition of the TNM staging system (Figures 8–11).

## 4. Discussion

Previously, tumor progression was thought to be related to genetic background. An increasing number of studies have demonstrated the key role of inflammation in tumor cell survival and proliferation, novel angiogenesis, apoptosis resistance, immune evasion, metastasis to distant regions and metastasis, and therapy resistance (23). Moreover, some studies have demonstrated that nutritional status plays a decisive role in tumorigenesis and progression. Therefore, the effects of inflammation and nutrition in patients with cancer, including diffuse large B-cell lymphoma (15), osteosarcoma (24) and pancreatic cancer (25).

We collected data on pre-treatment systemic inflammatory and nutritional markers (including BMI, PNI, and NLR) in patients with NPC to examine their clinical and prognostic value and to compare their predictive accuracy. To our knowledge, this is the first study to investigate the prognostic value of inflammatory and nutritional indicators in patients with NPC based on the 8th edition of the AJCC staging system. Our results showed that pre-treatment PNI and NLR had a significant impact on survival in patients with NPC. To effectively distinguish the two prognostic groups, we identified an optimal PNI cutoff point of 48.1. This value was comparable to those reported previously (26–28). Miao et al. (16) suggested that the PNI is an independent reliable prognostic factor in patients with NPC undergoing IMRT. In patients with stage II–IVb disease and PNI ≤ 52.0, CCRT alone did not provide satisfactory results, and additional treatments were required. That study was based on the 7th edition of the UICC/AJCC staging system. However, the 8th edition of the AJCC staging system for NPC has been released and is now widely used. Wei et al. (17) also observed a significantly lower OS in patients with a high PNI (>51) compared to that in patients with metastatic NPC. In their propensity score-matched analysis, Lin (29) confirmed that patients with non-metastatic NPC with PNI <51 were more likely to develop distant metastases. However, that study used the 7th edition of the AJCC/UICC TNM system for clinical staging and did not provide a detailed description of the radiotherapy technology.

Clinicians evaluate prognosis according to the TNM stage and then choose from among therapeutic options. Therefore, accurate staging is important. Although many studies have confirmed that the 7th edition staging system of NPC accurately predicts survival prognosis, and it has been widely internationally adopted (30, 31), this edition has ambiguous definitions that lead to challenges in clinical practice (32, 33). This version of the staging system is primarily based on studies using traditional 2D/3D techniques. However, the widespread use of IMRT and MRI have allowed significant improvements in the survival of patients with NPC, making the 7th edition outdated. Fortunately, these limitations improved in the 8th edition. To our knowledge, this is the first study on the prognosis of PNI in patients with NPC undergoing CCRT with or without AC based on the 8th staging system.

Recent studies have increasingly focused on the effect of nutrition and immune status on the prognosis of patients with cancer (8, 9, 11, 34). The value of NLR in the prognosis of NPC has been supported by many reviews (35–37). The prognostic value of PNI has also been demonstrated in various malignancies (10, 11, 25, 38). However, few studies have determined which prediction value is greater. Lin (29) reported that the PNI had a better discriminatory ability for predicting 1-, 3-, and 5-year DMFS than other inflammatory scores. The results of the present study also showed better discernment of the PNI in predicting the 5-year OS than the NLR. Several indicators of inflammation were included (NLR and PNI); however, only the PNI was an independent prognostic factor for locally advanced NPC. Therefore, the PNI may represent a novel prognostic factor for patients with NPC, which is based on inflammation and nutrition. PNI is an independent prognostic factor even in middle-aged and elderly patients with non-metastatic NPC (22). Although the pre-treatment PNI is an excellent prognostic marker for NPC patients, the post-treatment PNI is also an independent prognostic marker for OS. PNI dynamics are independent prognostic indicators for OS (21).

The PNI can predict tumor survival because (1) lymphocytes are the major component of participants in the immune response and play an important role in inhibiting tumor cell proliferation and metastasis (39). Lymphocyte counts represent the strength of the immune system; the lower the lymphocyte count, the weaker the systemic immunity. Thus, cancer cells easily evade immune surveillance, which augments their activity as cancer cells. (2) Serum albumin level is the most direct indicator of nutritional status in the human body. Albumin has been linked to tumor necrosis because pro-inflammatory cytokines reduce albumin production (38).

In this study, LDH was significantly associated with OS in univariate analysis (*p* = 0.032). These results are consistent with those previously reported. Xiong (40) observed that pretreatment PNI and LDH were not only statistically significantly associated with survival prognosis in patients with locally advanced NPC. Moreover, their combination, superior to individual scores, complemented the traditional TNM staging systems. The results of a meta-analysis of 18 studies, including 13,789 patients, suggested that high serum LDH levels are associated with worse outcomes in patients with NPC (41). Although numerous studies have confirmed the prognostic value of LDH, the molecular mechanisms linking LDH to distant metastasis remain unclear (42–44). Firstly, it is thought to be linked to glycolysis in cancer cells, which utilize different metabolic pathways than normal cells. Despite the presence of oxygen, cancer cells preferentially utilize the anaerobic glycolysis pathway to produce energy, a phenomenon histologically known as the Warburg effect (45). In anaerobic environments, LDH promotes the conversion of pyruvate to lactic acid, which plays a key role in anaerobic glycolysis. As large amounts of lactic acid are produced, adjusting LDH levels upward ensures efficient activity. Second, the immunosuppressive effect of lactic acidosis is strong enough to mediate tumor immune evasion (46). In addition, in many cases, tumor tissues show extensive cellular damage and release more extracellular enzymes than normal tissues, including LDH (47).

Numerous nomograms or prognostic models have been developed for NPC, but few clinicians have used them in the clinic. There are several reasons for this: (1) Some prognostic models incorporate indicators that are not routinely used in clinical practice, such as C-reactive protein (CRP) (48), high-sensitivity-CRP (hs-CRP) (49), and positron emission tomography (PET)/CT (50), and have limitations in clinical promotion. (2) Some models are based on previous old staging systems (51), such as the UICC2002 TNM stage system, and the latest edition staging system has been widely adopted in clinical practice. (3) Some prognostic models include patients treated with mixed radiotherapy techniques, including intensity-modulated and two-dimensional radiotherapy techniques (48, 49). (4) Some models have not been externally validated (52). Formally, because current prognostic models are more or less flawed, new prognostic models are constantly being developed to improve and modify them. The search is ongoing for the best and most clinically applicable models that can be widely used. We constructed a nomogram based on inflammatory biomarkers and nutritional indicators (53), including Epstein–Barr virus (EBV)-DNA. However, this is not a routine screening program in some centers, and not all patients have these data available, particularly in some basic hospitals. Furthermore, differences in the standards for EBV-DNA assays. All these factors have limited clinical applications. Therefore, we combined the factors identified in this study (age, PNI, and LDH) to develop and validate a nomogram to predict OS in patients with NPC that is more practical in the clinical setting. The nomogram showed a more accurate prognostic capability compared to the conventional TNM staging system, which is anatomically informative and does not consider tumor heterogeneity. The individualized stratification and precise treatment of tumors have some deficiencies. We used the C-index and AUC to compare the accuracy of nomogram and TNM staging system predictions. The nomogram and TNM staging system had C-indices of 0.702 and 0.647, respectively. The nomogram had a higher AUC than that of the TNM staging system. In addition to the C-index and AUC, we used AIC and DCA to compare the two models. The lower the AIC, the higher the discriminatory ability of the model. In our study, the nomogram was associated with a lower corrected AIC (1142.54) compared to the TNM staging system (1163.70). Moreover, the results of the decision curve analysis showed that the nomogram had a higher net benefit in predicting OS clinical utilization compared to the 8th edition TNM staging system. Thus, our results demonstrate the better prediction accuracy of the nomogram.

The present investigation has several limitations. First, this retrospective study was conducted at a single center. Second, we could not examine other markers such as EBV-DNA and CRP. Moreover, because the related data were insufficient or inappropriate, we were unable to analyze them. Although plasma EBV-DNA is an important marker of survival and has been confirmed in numerous studies (54–56), the current use of plasma EBV-DNA has some limitations. (1) Not all patients with pathologically confirmed NPC have detectable plasma EBV-DNA; moreover, studies have shown that even in endemic areas, plasma EBV-DNA is still undetectable in 12–29% of confirmed cases at initial diagnosis (57–59). (2) Owing to the wide variation in copy number determined by quantitative assays of EBV-DNA in plasma performed in different clinical laboratories, there remains no consistent standard or standardized method of analysis. The EBV-DNA copy number is highly variable between laboratories, and there remains no uniform standard. (3) The lowest detection limit also varies widely among different institutions, resulting in variations in false-negative rates and recommended cutoff values. Only 28.4% of the journal articles clearly stated the lower detection limit of their EBV-DNA assays, ranging from 0 to 1,000 copies/ml (60). The recommended cut-off value for classifying patients into different risk groups varies considerably, from 500 to 4,000 copies/ml (61, 62). Thus, there is a need for the standardization of EBV-DNA serological assays to allow the comparison of results between different laboratories and populations in different translational studies (63). Third, this study only explored the relationship between PNI and prognosis at a single pre-treatment time point and did not address the relationship between PNI kinetics and prognosis. Finally, validation in a single institution has certain limitations; therefore, validation in external and prospective populations is required.

## Data availability statement

The original contributions presented in the study are included in the article/supplementary material, further inquiries can be directed to the corresponding author/s.

## Ethics statement

This study was approved by the Ethics Committee of Guangxi Medical University Cancer Hospital (LW2022029), in compliance with the Declaration of Helsinki. The patients/participants provided their written informed consent to participate in this study.

## Author contributions

Y-MJ and X-DZ studied conception and design. Y-MJ, X-BP, and J-LM: data acquisition. X-BP and S-TH: data analysis and interpretation. S-TH and J-LM: quality control of data and algorithms. Y-MJ: manuscript writing. X-BP, S-TH, and X-DZ: manuscript reviewing and approving. All authors contributed to the article and approved the submitted version.

## Funding

This work was supported by the Health Commission of Guangxi Zhuang Autonomous Region (no. Z-A20220684).

## Conflict of interest

The authors declare that the research was conducted in the absence of any commercial or financial relationships that could be construed as a potential conflict of interest.

## Publisher’s note

All claims expressed in this article are solely those of the authors and do not necessarily represent those of their affiliated organizations, or those of the publisher, the editors and the reviewers. Any product that may be evaluated in this article, or claim that may be made by its manufacturer, is not guaranteed or endorsed by the publisher.
